# Esophageal Schwannoma: A Rare Benign Esophageal Tumor

**DOI:** 10.7759/cureus.15667

**Published:** 2021-06-15

**Authors:** Rasiq Zackria, Eric H Choi

**Affiliations:** 1 Internal Medicine, University of California, Riverside - School of Medicine, Riverside, USA; 2 Gastroenterology, Riverside Medical Clinic, Riverside, USA

**Keywords:** endoscopic ultrasound (eus), esophageal tumor, benign pathology, schwannoma, immunohistochemistry staining, endoscopy

## Abstract

Benign primary tumors are uncommon, with the majority of these tumors being leiomyomas; schwannomas of the esophagus are rare. Here, we present a case of a 78-year-old woman referred for complaints of intermittent dysphagia with a chest computed tomography scan showing a homogenous mass, compressing the esophagus. Upper gastrointestinal endoscopy revealed a submucosal mass, which was eventually diagnosed as a schwannoma after an endoscopic ultrasound with fine-needle aspiration and subsequent pathologic and immunohistochemical examination. Schwannomas could be managed conservatively.

## Introduction

Schwannoma is a tumor of the peripheral nervous system. It originates in Schwann cells and usually refers to a benign, slow-growing tumor [1]. While this type of tumor is found frequently in the head, neck, and extremities, it is rarely found in the gastrointestinal tract [1]. Esophageal tumors are uncommon, with malignant tumors being predominant. Benign tumors represent approximately 2% of all esophageal tumors [2]. Most are leiomyomas, while schwannomas of the esophagus are rare. These tumors tend to occur in the upper and mid-esophagus. Esophageal obstruction due to a schwannoma has not been previously reported, with most cases describing tracheal compression [2]. While dyspnea has been the primary reported symptom, dysphagia has been rarely reported. These tumors were thought to be a diagnostic challenge, and surgical resection was needed to confirm the diagnosis. However, the advancement in endoscopy has made diagnosing easier. In this case report, we describe our endoscopic experience of a case of an older woman presenting with dysphagia who was subsequently diagnosed with an esophageal schwannoma.

## Case presentation

**Figure 1:**
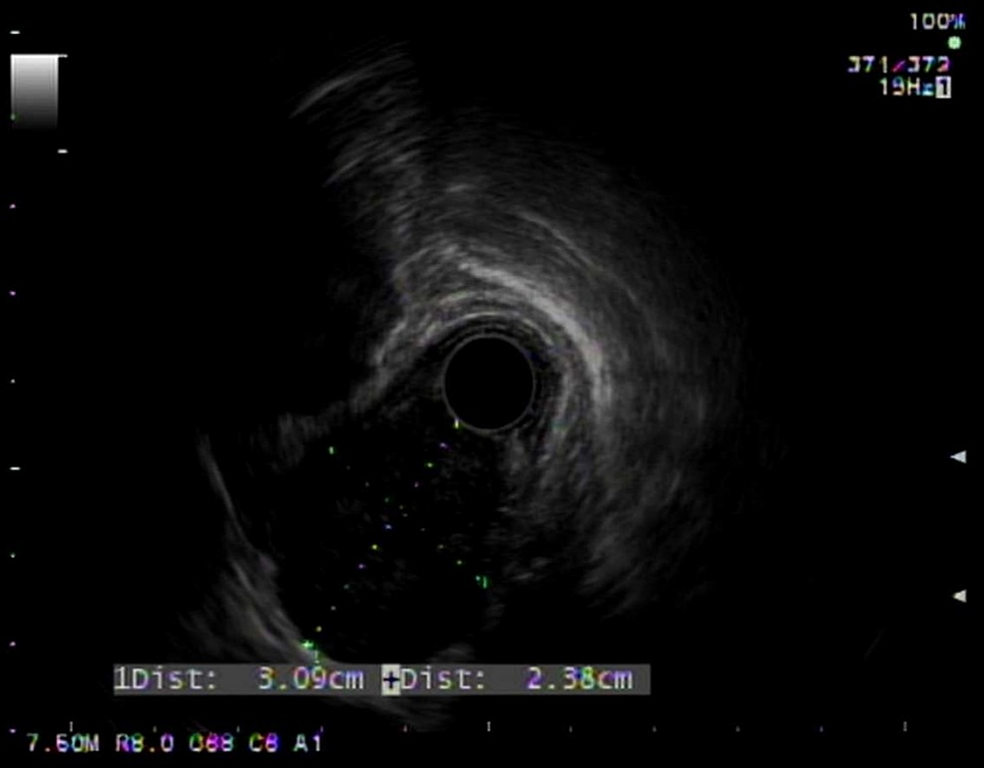
Initial endoscopic ultrasound view of the 3 cm hypoechoic lesion.

**Figure 2:**
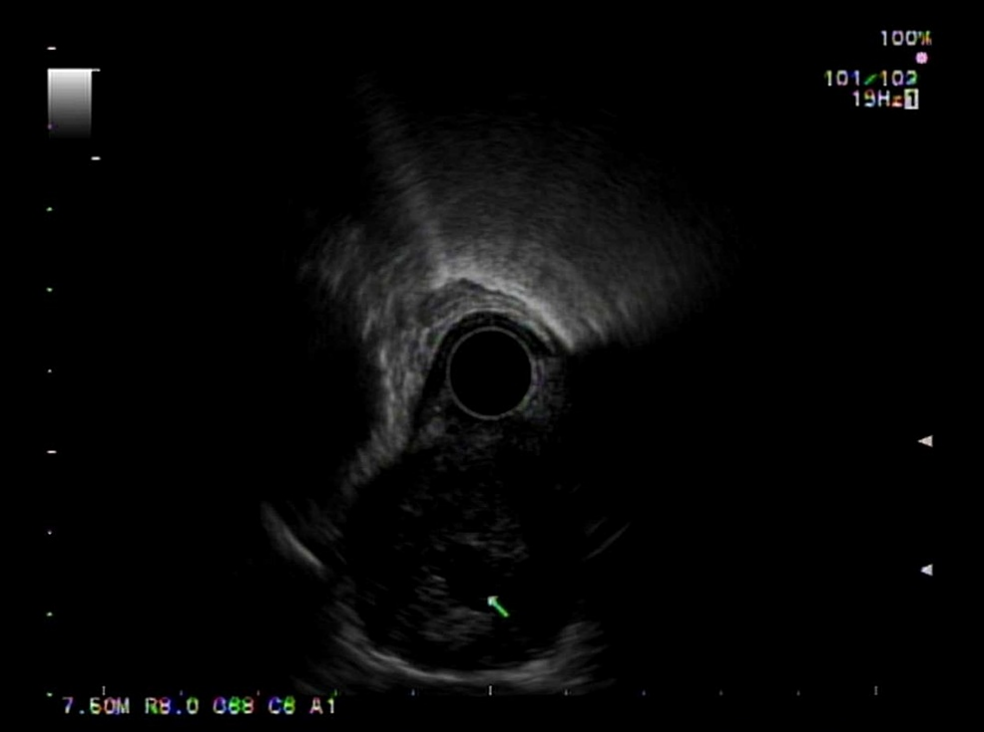
Endoscopic ultrasound view of the esophageal schwannoma hypoechoic lesion with anechoic cystic space.

A 78-year-old woman was referred for intermittent dysphagia for the past year. She denied any medical history and reported that dysphagia was present in both solids and liquids. She did not seek medical attention earlier due to the intermittent nature of her symptoms. She denied unintentional weight loss, hematemesis, dyspepsia, or reflux. Furthermore, she denied a family history of malignancy. Physical examination was unremarkable. A computed tomography scan of the chest showing a 3 cm, well-demarcated, enhancing, homogenous mass compressing the esophagus. Endoscopic evaluation showed a submucosal mass in the middle third of the esophagus with endoscopic ultrasound (EUS) demonstrating a 3 cm hypoechoic lesion with numerous anechoic spaces, consistent with cystic spaces (Figures 1, 2).

**Figure 3:**
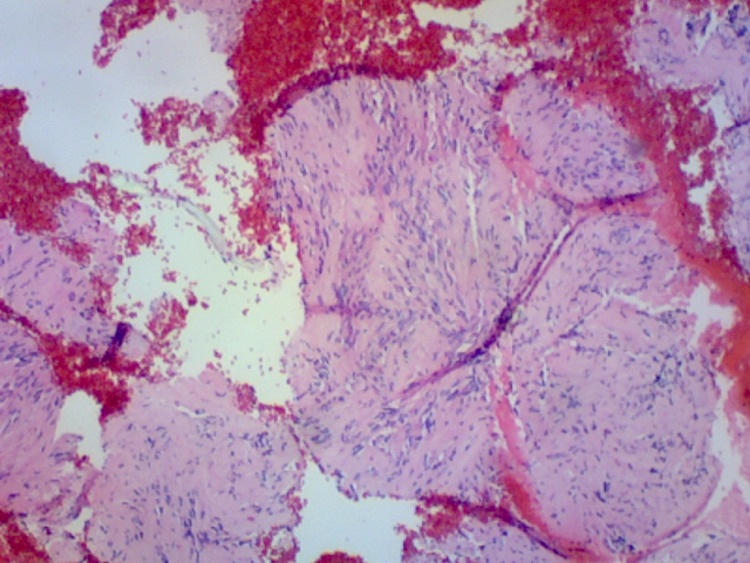
H&E stain demonstrating spindle cells with bland nuclei and ill-defined cytoplasm. Located between the spindle cells are Verocay bodies. H&E: hematoxylin and eosin.

Fine-needle aspiration (FNA) demonstrated spindle cells surrounding acellular areas consistent with Verocay bodies on hematoxylin and eosin (H&E) stain (Figure 3), and immunohistochemical markers were negative for CD-117, DPG-1, desmin, and calponin, decreasing the possibility of a gastrointestinal stromal tumor or leiomyoma. 

**Figure 4:**
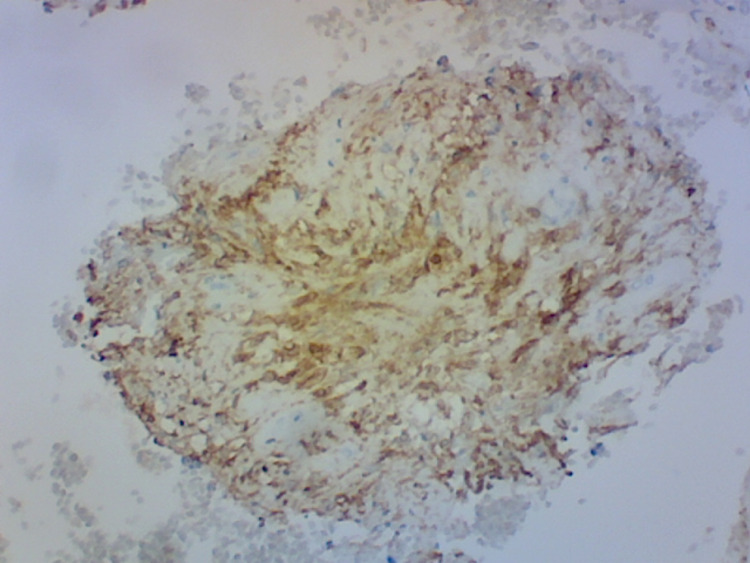
Immunostain with S-100 protein positivity, confirming the diagnosis of benign esophageal schwannoma.

SOX-10 and S-100 immunostaining markers showed positive results suggesting a neural origin of the tumor consistent with the diagnosis of esophageal schwannoma (Figure 4).

The patient’s preference was for conservative surveillance with the possibility of surgical intervention, if symptoms were to progress.

## Discussion

Esophageal tumors are primarily malignant with benign esophageal neoplasms representing approximately 2% of all esophageal tumors [2]. Leiomyomas are the most common of these benign tumors, while esophageal schwannomas are rarely reported [2,3]. Due to their featureless and nonspecific characteristics on imaging studies, it can be challenging to differentiate esophageal schwannomas from other intramural esophageal lesions. Most patients are asymptomatic and incidentally found to have benign esophageal tumors. While the most common symptom is dyspnea, dysphagia has been reported in some cases [2,4]. Esophageal obstruction from schwannomas has not been reported, and surgical resection is typically unnecessary [5]. However, surgical treatment should be considered for benign esophageal tumors that are large, symptomatic, or increasing in size [6].

While esophageal schwannomas are benign tumors, they show a hypermetabolic appearance on 18-fluorodeoxyglucose positron emission tomography, similar to findings of malignant esophageal tumors [6]. Due to the limited accuracy of this technique, endoscopic ultrasound-guided FNA can be used to aid in establishing the pathologic diagnosis [6]. As esophageal masses cannot be diagnosed as benign or malignant based on imaging or endoscopy, all esophageal masses encountered should be biopsied. The parameters of schwannoma immunohistochemistry are based on positivity for S-100 and SOX-10 protein; other classic markers such as CD34, CD117, desmin, and caldesmon (suggestive of gastrointestinal stromal tumors or leiomyoma) are virtually negative [3].

Once pathologic diagnosis is established, endoscopy +/- ultrasound for periodic surveillance has been suggested for asymptomatic lesions < 2cm in diameter, as these lesions often tend to be benign. Further interventions to evaluate and resect the lesion can be considered if any changes are encountered on surveillance. Endoscopic treatment may be attempted for small tumors, while enucleation by a thoracoscopic approach and esophagectomy may be considered for those lesions not amenable to endoscopic resection.

## Conclusions

Endoscopic ultrasound has been increasingly used to diagnose (when done in conjunction with FNA) and monitor esophageal tumors; it is the most accurate imaging modality to determine the locoregional spread of esophageal malignancy. Esophageal schwannoma is rare and should be considered in the differential when a submucosal esophageal mass is encountered. While complete excision of the mass for large lesions is widely performed, conservative management can be considered for minimally symptomatic patients, as in our case. 
